# The Effects of Mind-Wandering, Cognitive Load, and Task Engagement on Working Memory Performance in Remote Online Experiments

**DOI:** 10.1027/1618-3169/a000599

**Published:** 2024-01-30

**Authors:** Kelly Cotton, Joshua Sandry, Timothy J. Ricker

**Affiliations:** ^1^ Department of Neurology, Albert Einstein College of Medicine, Bronx, NY, USA; ^2^ Department of Psychology, Montclair State University, Montclair, NJ, USA; ^3^ Department of Psychology, University of South Dakota, Vermillion, SD, USA

**Keywords:** working memory, short-term memory, mind-wandering, complex span, multitasking

## Abstract

**Abstract:** Recent changes in environments from in-person to remote present several issues for work, education, and research, particularly related to cognitive performance. Increased distraction in remote environments may lead to increases in mind-wandering and disengagement with tasks at hand, whether virtual meetings, online lectures, or psychological experiments. The present study investigated mind-wandering and multitasking effects during working memory tasks in remote and in-person environments. In two experiments, participants completed a working memory task with varied cognitive load during a secondary task. After each working memory trial, participants reported their mind-wandering during that trial. Some participants completed the procedures in-person, while others completed the procedures remotely. Overall, remote participants reported significantly more mind-wandering and poorer secondary task performance than in-person participants, but this pattern was not reflected in working memory accuracy. Both groups exhibited similar multitasking effects on performance. Additional analyses found that for remote participants, task engagement better predicted working memory performance than either cognitive load or mind-wandering rates but did not indicate a tradeoff in resources between tasks. Together, these results demonstrate the importance of considering multiple metrics when assessing performance and illustrate that making assumptions about the equivalence of remote and in-person work is a risky proposition.


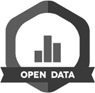


Recently, there has been an accelerated shift in education, work, and scientific research to remote online environments. Many have assumed that cognitive performance in online and in-person environments is at most, trivially different. This belief is supported by research suggesting that performance on some cognitive tasks is similar across contexts (Barnhoorn et al., 2015; Crump et al., 2013; Hilbig, 2016; Semmelmann & Weigelt, 2017; Uittenhove et al., 2023); however, this shift in environments may have yet unidentified effects on more complex cognition, particularly under less-than-ideal conditions. For example, out-of-lab environments are likely to have considerably more distractors compared to an in-lab environment. In turn, this distraction may contribute to performance detriments during difficult tasks that require sustained working memory resources. Working memory, the system for holding a limited amount of information in attention to be used in ongoing cognition (Baddeley & Hitch, 1974; Cowan, 1988), is involved with many higher order cognitive processes (Daneman & Carpenter, 1980; Engle et al., 1999; Hitch, 1978; Süß et al., 2002; Unsworth et al., 2009), so it is particularly pertinent to understand how it functions across different environments.

In daily life, we are rarely afforded the opportunity to engage with a single cognitive task as is common in laboratory settings. Instead, we often must juggle concurrent tasks with varying demands. For example, when attempting to remember a phone number, you may have to move to a new room and locate your phone, ignore other notifications, and navigate to the correct screen, all while maintaining several digits in working memory. There are several proposed explanations for the deleterious effects of multitasking on working memory, such as task-switching-related interference (Kiesel et al., 2010; Pettigrew & Martin, 2016; Vandierendonck et al., 2010) and most importantly to the present study, cognitive load (Barrouillet et al., 2004; Lemaire & Portrat, 2018; Oberauer & Lewandowsky, 2011; Oberauer et al., 2012; Portrat & Lemaire, 2015). Cognitive load refers to a measure of secondary task disruption in working memory and has been shown to impair working memory performance as it reduces the time available for maintenance operations relative to the total forgetting occurring (Barrouillet et al., 2004, 2007). In essence, as the difficulty of the secondary task increases, performance on the primary task decreases due to reduced opportunities for working memory maintenance. Individuals in an online environment may exhibit greater working memory impairment as distractors in the environment may shift attention away from working memory maintenance, much like increasing the cognitive load of a secondary task. If individuals can suppress distractors and stay on-task, then there should be no difference in performance online compared to in-person.

However, the ability to stay on-task may vary across environments. Mind-wandering refers to shifting attention away from a given task (Smallwood & Schooler, 2006) and may occur at varying rates depending on environment. Risko et al. (2013) compared how often participants reported mind-wandering while watching video lectures in two conditions, either alone or in a classroom with other students. The researchers found that participants mind-wandered at similar rates in both contexts, approximately 40% of the time. However, fully online studies have found that participants watching video lectures reported task-related interference (i.e., “how well I’m understanding the video”) and task-unrelated thoughts more often (63%, Hollis & Was, 2016; 76%–81%, Was et al., 2019). A recent study compared mind-wandering rates across contexts during a sustained attention task in younger and older adults, finding that both groups reported mind-wandering less often at home compared to in an in-person lab setting (Diede et al., 2022). The authors suggest that the relative lack of external stimuli in a lab setting leads to higher rates of mind-wandering. Additionally, in a meta-analysis consisting of nearly 3,000 participants, Drody et al. (2023) found that online participants reported engaging in distracting media multitasking 38% of the time, with some studies reporting higher rates up to 85% of the time. Together, these results suggest that while even in-person settings can induce mind-wandering, online environments may exacerbate this effect.

While previous research has investigated the rates of mind-wandering while completing a relatively simple task, passively watching videos or sustained attention tasks, mind-wandering has also been shown to impair performance on several more challenging cognitive tasks (for review, see Mooneyham & Schooler, 2013). In particular, it has a marked negative effect on working memory performance (Banks & Boals, 2017; Banks et al., 2015; Krimsky et al., 2017; Mrazek et al., 2012). Mrazek et al. (2012) examined the relationship between working memory capacity and mind-wandering on a trial-by-trial basis in an experiment using a complex span task. Participants were asked to indicate if their attention was on task-related or task-unrelated thoughts on 60% of the trials. The researchers found that greater overall mind-wandering was associated with poorer working memory performance and that participants mind-wandered more during inaccurate trials than during accurate trials. Furthermore, there was a greater effect of mind-wandering for the large set size compared to smaller size trials. Similarly, Krimsky et al. (2017) investigated how time on task may influence mind-wandering during a complex working memory task. The researchers found that when participants reported mind-wandering, working memory accuracy dropped. Additionally, they found that mind-wandering increased over time, particularly for high load trials, while working memory accuracy decreased. These results suggest that while the effects of mind-wandering on cognitive performance are often detrimental, they are not uniform and may depend on several factors, including task difficulty or time on task.

The present study aimed to investigate cognitive load, mind-wandering, experimental context, and their combined effects on working memory performance. There are several potential outcomes to this study. First, on the basis of existing research (Barrouillet & Camos, 2012; Barrouillet et al., 2004, 2007; Plancher & Barrouillet, 2013), increased cognitive load should lead to performance impairments in both online and in-person participants. Furthermore, online participants may perform worse than in-person participants on the primary working memory task and report higher rates of mind-wandering. This would suggest that the mind-wandering caused by a more distracting environment is adding to the cognitive load of the task. A second outcome is that online participants will show no performance differences, but they may report higher mind-wandering rates than in-person participants. Such a finding would suggest that environmental context alters mind-wandering, but this mind-wandering does not increase the cognitive load of a task or affect subsequent working memory performance. A third potential outcome is that, while increased mind-wandering due to higher cognitive load may lead to worse memory performance, this effect may not change across experimental contexts. These results would indicate that the online environment is not inherently any more distracting than the in-lab environment and that experimental context may not matter for tests of working memory. Finally, there may be no interactive effect between cognitive load, mind-wandering, and experimental context on working memory performance. For example, recent research has questioned the universality of cognitive load effects and demonstrated boundary conditions, with the robustness of cognitive load theory only applicable in limited circumstances (Ricker & Vergauwe, 2020, 2022). Therefore, variation in working memory performance may not be due to cognitive load and mind-wandering but rather some other factor. Regardless of outcome, exploring memory creation across various contexts will improve our understanding of human cognition and its processing limits and is a necessary first step in developing strategies to mitigate the detriment on task performance.

## Experiment 1

The goal of Experiment 1 was to investigate whether higher cognitive load during a complex working memory task leads to increased mind-wandering, particularly when the task is completed online rather than in-person. Participants completed a complex working memory task which varied the amount of cognitive load during the task. On each trial, participants were asked what they were thinking about, among several options. Two different groups of participants completed the task procedures either online or in-person. This design allows us to investigate the effects of both cognitive load and mind-wandering on working memory performance and how these effects differ across experimental contexts.

### Methods

#### Participants

Of 185 participants, 132 participants (*M*_age_ = 19.7, 80% female, 20% male) completed the study procedures online and 53 participants (*M*_age_ = 19.8, 51% female, 44% male, 5% other or unknown) completed the study procedures in-person. All participants self-selected to complete the procedures online or in-person, and no participant completed both versions. One online participant reported colorblindness and was removed the analyses, for a total of 184 participants. All participants were students enrolled in undergraduate psychology courses who participated in exchange for partial course credit. These experiments were approved by the Institutional Review Boards at the University of South Dakota and Montclair State University. Informed consent was obtained from all individual participants included in the study.

#### Materials

Mind-wandering was queried by asking participants what they were thinking about during the preceding trial. The eight categorical options are adapted from Kane et al. (2021) and included *the task*, *task experience/performance*, *everyday things*, *current state of being*, *personal worries*, *daydreams*, *external environment*, or *other*. Subjective experience of everyday mind-wandering was assessed with the Mindfulness Attention Awareness Scale (Brown & Ryan, 2003).^1^ The experimental paradigm was programmed using PsychoPy software (Peirce et al., 2019) and run locally (in person) or over the Internet browser hosted on Pavlovia.

#### Procedure

**Figure 1:**
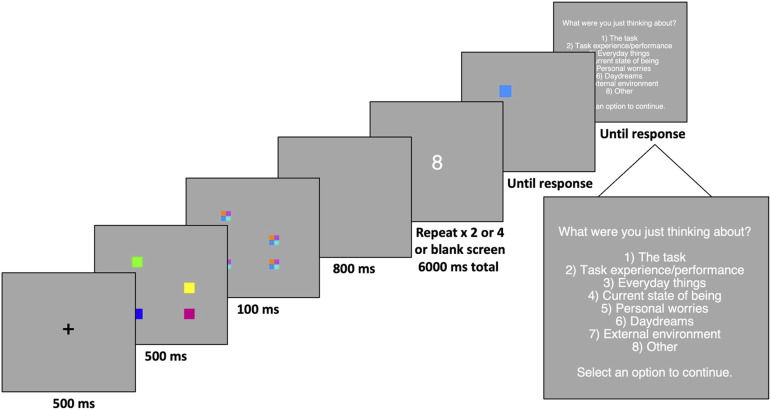
Procedure for a typical trial in Experiment 1.

Participants first completed a short demographic questionnaire, followed by a short practice session before beginning the visual working memory task. At the beginning of each trial of the working memory task, a fixation cross was presented for 500 ms. Participants were then presented with four colored squares for 500 ms followed by visual masks (consisting of jumbled colored squares in the same location as the memory items) and a blank delay interval of 800 ms. Participants then completed the secondary task, during which a single digit was presented, and participants indicated by button press if the number was odd or even. The total length of the secondary task was six seconds, and the cognitive load was manipulated by varying the number of secondary task items presented (zero, two, or four items). After completion of the secondary task, participants were then presented with one colored square in the same location as a previous memory item and asked to indicate if the color of the square had changed. During *change* trials, the color of the square at test was a completely new color and had not been presented in the earlier array. After each trial, participants were asked about mind-wandering during the preceding trial. In response to the question “What were you just thinking about?”, participants indicated via button press which categorical response best fits what they were thinking about. After responding to the mind-wandering question, a new trial began. In total, participants completed 144 working memory task trials. There were an equal number of change and *no-change* trials. A single trial is depicted in Figure 1. After completion of all trials, participants completed the Mindful Attention Awareness Scale.

#### Data Analysis

Prior to conducting our analyses, we excluded trials in which the reaction time to the primary working memory task was less than 300 ms (0.7% of all trials). Additionally, two online participants were missing mind-wandering probe data for one trial each. These trials were removed from our analyses.

For our analyses related to performance on the primary working memory task, we calculated the proportion of trials that were correctly identified as either a *same* trial or a *different* trial for each participant. For our analyses related to mind-wandering, we calculated the proportion of trials in which each participant selected any option other than the task, such that a higher value indicates a greater proportion of trials spent mind-wandering. Finally, for our analyses related to performance on the secondary parity judgment task, we calculated the proportion of trials in which the number was correctly identified as either *even* or *odd*. Importantly, because the length of the secondary task was fixed rather than based on the participants' response time, it was possible for a participant not to respond to one or more of the secondary task items during any given trial. Missing responses were coded as incorrect in the present analyses.

We computed Bayes factors for *t* tests (Rouder et al., 2009) and analysis of variance (ANOVA) effects (Rouder et al., 2012) using the BayesFactor package v0.9.12-4.3 (Morey & Rouder, 2018) in R statistical computing software (R Core Team, 2019). We used the default settings of the package with an effect size *SD* of (√2)/2. Bayes factors indicate the probability of the data assuming an effect of the manipulation relative to the probability of the data when assuming no effect is present. For example, a Bayes factor of 10 in favor of an effect indicates that the data are 10 times more likely under the alternative hypothesis relative to the null hypothesis. Bayes factors greater than 3 are typically interpreted as providing substantial evidence.

### Results

#### Primary Working Memory Task Accuracy

**Figure 2:**
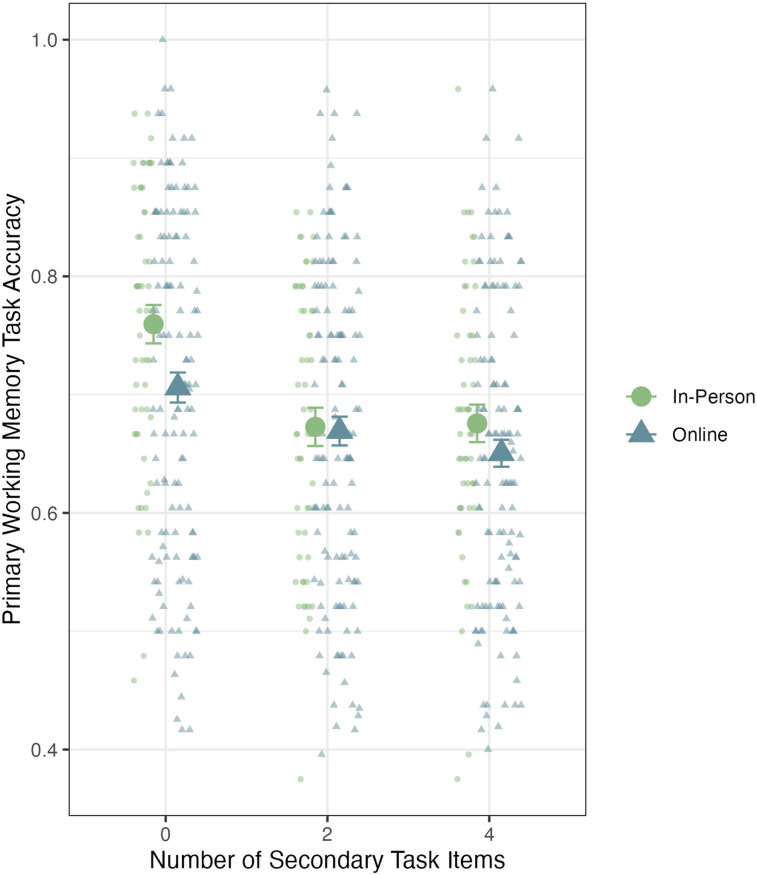
Working memory accuracy across cognitive load conditions for in-person and online participants. Small points represent individual participant performance, and the large points represent the group performance. Error bars represent standard error of the mean.

A 2 (Experimental Context: Online vs. In-Person) × 3 (Cognitive Load: 0, 2, or 4 secondary task items) Bayesian ANOVA for working memory accuracy found ambiguous evidence against a main effect of experimental context, Bayes factor = 1.3 in favor of a null effect, and strong evidence to support a main effect of cognitive load, Bayes factor = 819 in favor of an effect. We found no evidence of an interaction effect, Bayes factor = 15 in favor of a null effect. These results are depicted in Figure 2. We conducted follow-up Bayesian *t*-tests to investigate which levels of cognitive load in the secondary task (0, 2, or 4 items) showed different performance. We found strong evidence for a difference between the 0-item condition and both the multitasking conditions (two items, Bayes factor = 2.5 × 10^9^ in favor of an effect; four items, Bayes factor = 1.8 × 10^13^ in favor of an effect). We found ambiguous evidence against a difference between the two-item and four-item multitasking conditions, Bayes factor = 1.9 in favor of a null effect.

#### Mind-Wandering Reporting

**Figure 3:**
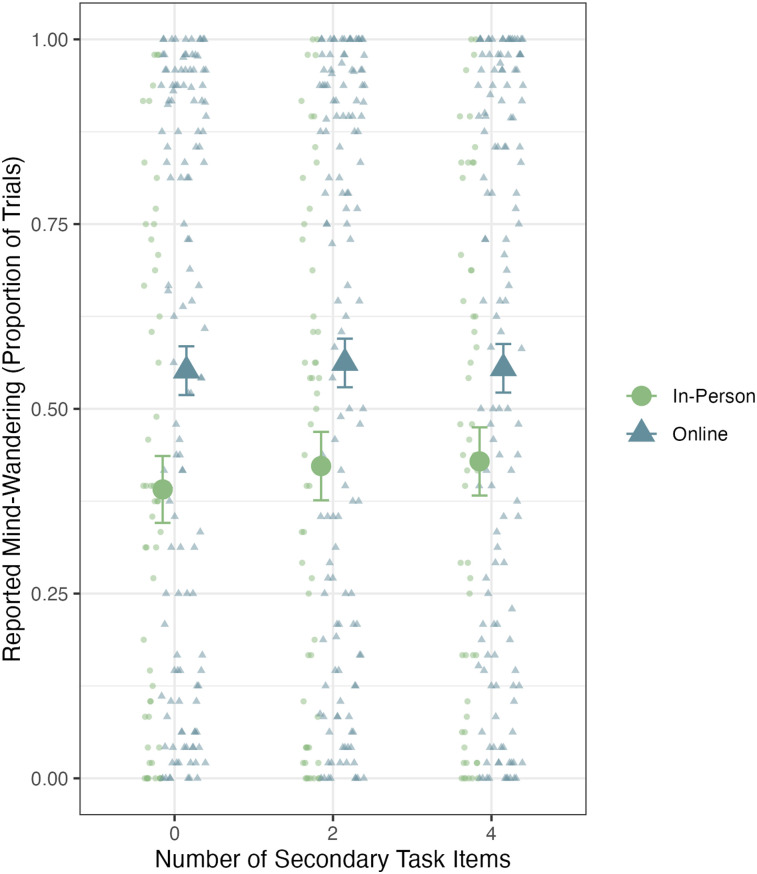
Reported mind-wandering across cognitive load conditions for in-person and online participants. Small points represent individual participant performance, and the large points represent the group performance. Error bars represent standard error of the mean.

To assess the effects on mind-wandering, we conducted a 2 (Experimental Context: Online vs. In-Person) × 3 (Cognitive Load: 0, 2, or 4 secondary task items) Bayesian ANOVA for proportion of trials in which participants reported mind-wandering. We found strong evidence to support a main effect of experimental context, Bayes factor = 321 in favor of an effect, but no main effect of cognitive load, Bayes factor = 84 in favor of a null effect. Again, we found no evidence of an interaction effect, Bayes factor = 3,441 in favor of a null effect. These results are depicted in Figure 3.

#### Secondary Task Accuracy

**Figure 4:**
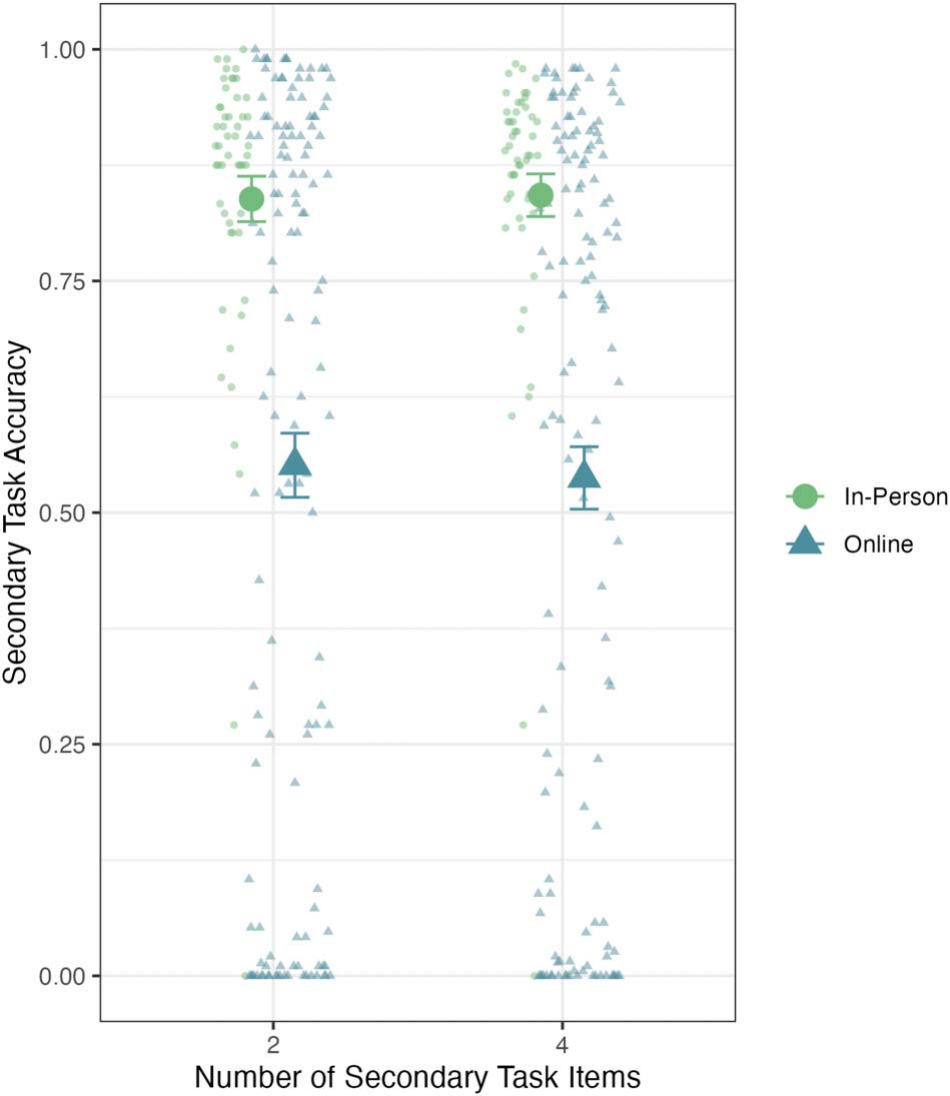
Secondary task accuracy across span conditions for in-person and online participants. Small points represent individual participant performance, and the large points represent the group performance. Error bars represent standard error of the mean.

We conducted a 2 (Experimental Context: Online vs. In-Person) × 2 (Cognitive Load: 2 or 4 secondary task items) Bayesian ANOVA for performance on the secondary task. We again found strong evidence to support a main effect of experimental context, Bayes factor = 1.5 × 10^10^ in favor of an effect, but no main effect of cognitive load, Bayes factor = 12 in favor of a null effect. We also did not find evidence of an interaction effect, Bayes factor = 98 in favor of a null effect. These results are depicted in Figure 4. When responses were incorrect for the in-person group (only 16% of responses), 40% were the wrong response and 60% were omissions. When responses were incorrect for the online group (46% of responses), 9% were the wrong response and 91% were omissions. This difference in incorrect and omission response rate across groups can also be considered in the context of all responses. For the in-person group, which overall made few errors, of all responses, 6.4% were the wrong response and 9.5% were omissions. For the online group, of all responses, 4.3% were the wrong response and 41% were omissions.

#### Mind-Wandering Reporting by Category

As previously discussed, online participants generally reported more mind-wandering compared to in-person participants. Until this point, we have operationalized mind-wandering as choosing anything other than the task when probed. However, there were several categorical options that participants could choose from to describe what they were just thinking about. Table 1 presents the percentage of responses to the thought probe for online and in-person participants. Online participants reported thinking about *the task* (44.3% of trials) less often than in-person participants (58.6% of trials). Furthermore, they also reported thinking about the task experience/performance (20.6% of trials) less often than in-person participants. Notably, among the other categories, online participants reported thinking about the external environment the most often (7.1% of trials), while this was the least reported option for in-person participants (1.5%).

**Table 1:** Responses to thought probe across all trials

Probe category	In-person (Experiment 1)	Online (Experiment 1)	Online (Experiment 2)
	%	%	%
The task	58.6	44.7	35.6
Task experience/performance	26.1	20.7	17.0
Everyday things	3.6	5.7	8.8
Current state of being	3.5	5.3	7.4
Personal worries	1.7	4.0	6.1
Daydreams	2.6	6.6	9.2
External environment	1.5	7.1	9.9
Other	2.4	5.9	6.0

### Discussion

The results of Experiment 1 demonstrate that online participants reported mind-wandering more often than in-person participants but perform similarly well on the primary working memory task. However, the online participants performed considerably worse on the secondary task. Notably, we found that several participants did not engage with the secondary task at all, resulting in a mean 0% accuracy, rather than the expected 50% accuracy if they were simply guessing. These errors were overwhelmingly driven by a failure to engage with the secondary task at all, as demonstrated by error responses resulting from an omission 91% of the time (41% of all responses) in the online group. Furthermore, while we found evidence of a general multitasking effect, this was seemingly unrelated to the cognitive load of the task, as increasing the cognitive load of the secondary task did not decrease primary task performance.

## Experiment 2

Experiment 1 found similar performance on the primary working memory task between online and in-person participants, despite higher rates of mind-wandering and worse secondary task performance. Importantly, working memory performance, mind-wandering rates, and secondary task performance did not share relationships with increasing cognitive load of the secondary task. The goal of Experiment 2 was to replicate these findings in our online participant group using one of the most common tasks for examining multitasking effects on working memory performance. Experiment 1 used a Brown–Peterson task to investigate this question; however, recent work suggests that the type of task may influence the effects of cognitive load (Ricker & Vergauwe, 2022). To increase the generalizability of the results of Experiment 1, we chose to use another working memory task, the complex span task, in Experiment 2. Participants completed a complex span working memory task which again varied the cognitive load of the secondary task to increase the cognitive load of the task. As in Experiment 1, participants also reported their thought focus during each trial. In this experiment, we were focused on understanding the observed behavior of online participants and as such, all participants completed the procedures online.

### Methods

#### Participants

A total of 191 students (*M*_age_ = 19.9, 67% female, 33% male) enrolled in undergraduate psychology courses participated online in exchange for partial course credit or monetary compensation. This sample size was increased relative to the previous sample to give sufficient power to explore potential between-group differences as detailed in the combined results section. Two participants completed the experiment twice, and four participants reported colorblindness and were removed from the analyses, for a total of 185 participants in the final sample. These experiments were approved by the Institutional Review Boards at the University of South Dakota and Montclair State University. Informed consent was obtained from all individual participants included in the study.

#### Materials

All materials were the same as Experiment 1.

#### Procedure

**Figure 5:**
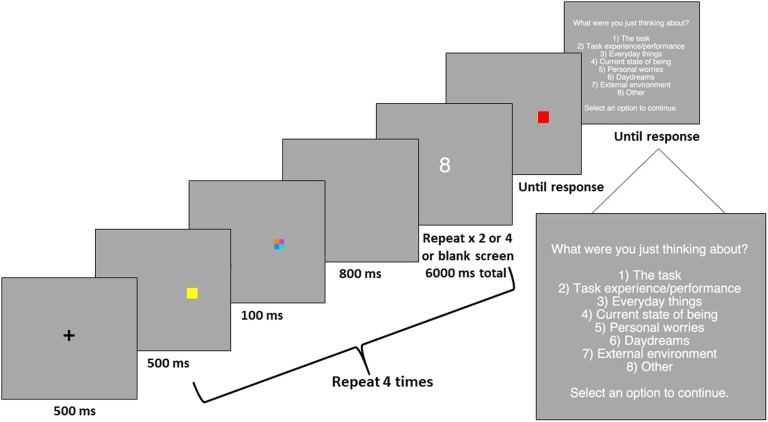
Procedure for a typical trial in Experiment 2.

The general procedure was the same as Experiment 1, except the memory items were presented as a complex span task. As in Experiment 1, the trial began with a fixation cross presented for 500 ms. Participants were then presented with a series of colored squares to remember. A single colored square was presented at a time in a random location for 500 ms followed by a visual mask (consisting of jumbled colored squares in the same location as the memory items) and a blank delay interval of 800 ms. Following each memory item, participants completed the secondary task, which was the same as in Experiment 1 (a two-item or four-item parity judgment task or a blank screen, 6,000 ms total). This sequence repeated for the next memory item and continued until all four memory items were presented. Participants were then shown a single colored square in the same location as a previously presented memory item and asked if the color of the square had changed. After their response, participants were asked about mind-wandering on each trial. After responding to the mind-wandering question, a new trial began. In total, participants completed 60 working memory task trials. There were an equal number of change and no-change trials. A single trial is depicted in Figure 5. After completion of all trials, participants completed the Mindful Attention Awareness Scale.^2^

#### Data Analysis

The analyses were the same as in Experiment 1. Again, we excluded trials in which the reaction time to the primary working memory task was less than 300 ms (0.3% of all trials).

### Results

#### Primary Working Memory Task Accuracy

**Figure 6:**
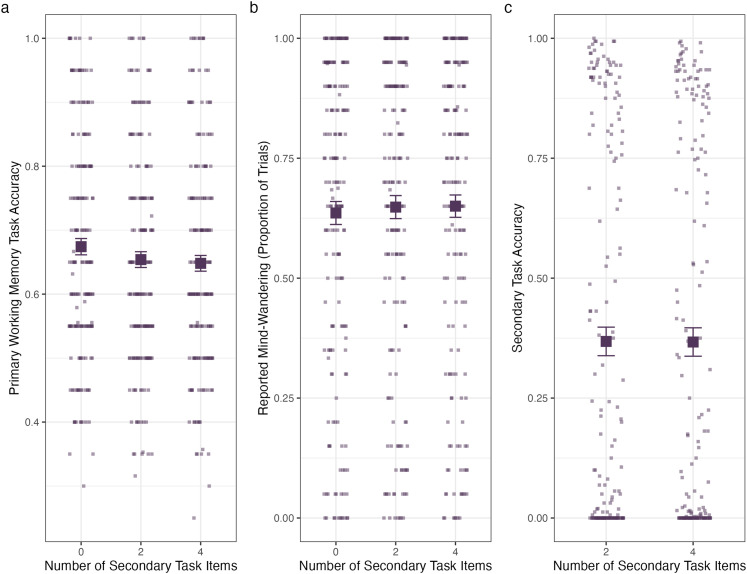
Performance across cognitive load conditions. Small points represent individual participant performance, and the large points represent the group performance. Error bars represent standard error of the mean. **A**. Working memory accuracy. **B**. Reported mind-wandering. **C**. Secondary task accuracy.

Overall, mean accuracy on the primary working memory task was .66 (*SD* = 0.47). A one-way (cognitive load: 0, 2, or 4 secondary task items) Bayesian ANOVA for working memory accuracy found evidence against an effect of cognitive load, Bayes factor = 30 in favor of a null effect. These results are depicted in Figure 6a.

#### Mind-Wandering Reporting

To assess the effects on mind-wandering, we conducted a one-way (cognitive load: 0, 2, or 4 secondary task items) Bayesian ANOVA for proportion of trials in which participants reported mind-wandering. Again, we found evidence against an effect of cognitive load, Bayes factor = 86 in favor of a null effect. These results are depicted in Figure 6b.

#### Secondary Task Accuracy

A Bayesian *t*-test for accuracy on the secondary task found evidence against a difference between the two-item and four-item conditions, Bayes factor = 11 in favor of a null effect. These results are depicted in Figure 6c. Of the errors that were made (63% of all trials), 5% were incorrect responses and 95% were omissions. When considered as a proportion of all responses, only 3.2% were incorrect responses while 60% were omissions.

#### Mind-Wandering Reporting by Category

As seen in Table 1 above, participants reported thinking about the task a little more than a third of the time, less than either group in Experiment 1. Again, we saw relatively high rates of thinking about the external environment (9.8% of trials).

### Discussion

As in Experiment 1, we did not find an effect of increasing cognitive load on working memory accuracy. Furthermore, while our sample reported high levels of mind-wandering overall, this did not vary with cognitive load nor did the relatively poor secondary task performance. Again, we found that many participants had a mean accuracy of 0% on the secondary task, suggesting that they were not engaging with the task entirely. Together with Experiment 1, these results suggest that online participants are either more resilient to cognitive load and mind-wandering effects on working memory or they are engaging with the task differently than in-person participants.

## Combined Analyses

As evidenced in Figures 4 and 6c, participants varied considerably on the secondary parity judgment task, particularly online participants. Notably, 40% of online participants in Experiment 1 performed below chance in the secondary task (.50 if we assume they responded on every trial), while only 4% of in-person participants performed this poorly. Furthermore, 15% of Experiment 1 online participants reported not engaging with the secondary task at all. This was not due to participants confusing the keys during the task (i.e., pressing the *O* key for even), but rather failing to respond at all. Averaged across all three groups, on trials in which participants failed to make a correct response, only 18% of the trials were an incorrect keypress, and the majority of incorrect trials (82%) were nonresponses. This pattern suggests that rather than participants trying to engage with the task but making errors, incorrect responses overwhelmingly reflected opting to not engage with the secondary task. However, this was not a consistent pattern across all participants, so in the next section, we aim to investigate how participants’ engagement with the overall task related to both working memory performance and mind-wandering in a larger combined sample.

As Experiments 1 and 2 had similar task designs, we pooled the data and divided the online participants into three groups based on secondary task performance: (1) high secondary task performance online participants (>80%, *n* = 105), (2) low secondary task online participants (≤80% and >0%, *n* = 137), and (3) zero secondary task performance online participants (0%, *n* = 74). The high/low secondary task performance threshold was chosen because it is a common performance criterion used to determine individual participant inclusion in working memory studies (e.g., Barrouillet et al., 2007; Portrat et al., 2008; Turner & Engle, 1989) and in line with our previous work (Cotton, Sandry, & Ricker, 2023). The zero-performance criterion was chosen to reflect complete disengagement with the secondary task as the only way to achieve this score was to not enter any responses. This analysis allows us to better understand how individual differences in task engagement map onto differences in working memory and mind-wandering processes as a function of in-person versus online testing environments. One potential outcome is that participants who are not engaging with the secondary task outperform those participants who do engage with the secondary task. As their attention is no longer divided between tasks, the nonengaged participants can fully devote their attentional resources to the primary working memory task. Another possibility is that participants who do not engage with the secondary task also have a lower performance on the primary working memory task. If this result coincides with increased levels of mind-wandering, then together these results suggest that being online does lead to both poorer performance and increased mind-wandering, counter to previous results (Diede et al., 2022; Uittenhove et al., 2023).

**Figure 7:**
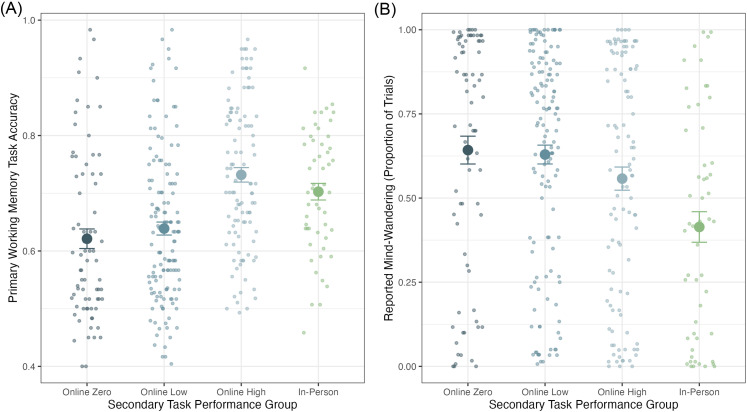
Performance comparing online secondary task performance groups and in-person participants. Small points represent individual participant performance, and the large points represent the group performance. Error bars represent standard error of the mean. **A** Working memory accuracy. **B** Reported mind-wandering.

For working memory accuracy, we found evidence supporting a difference between the in-person group (*M* = 0.70, *SD* = 0.11) and the zero performance online group (*M* = 0.62, *SD* = 0.15), Bayes factor = 39 in favor of an effect as well as ambiguous evidence supporting a difference between the in-person group and the low performance online group (*M* = 0.64, *SD* = 0.13), Bayes factor = 16 in favor of an effect. We did not find evidence that the high performance online group (*M* = 0.73, *SD* = 0.13) had higher working memory accuracy compared to the in-person group, Bayes factor = 2 in favor of a null effect. Finally, we found strong evidence that the high performance online group performed better on the working memory task compared to the other online groups (zero-performance online group, Bayes factor = 5.0 × 10^4^ in favor of an effect; low-performance online group, Bayes factor = 1.2 × 10^5^ in favor of an effect). These results are illustrated in Figure 7a.

For mind-wandering, we found evidence supporting a difference between the in-person group (*M* = 0.41, *SD* = 0.33) and both the zero-performance online group (*M* = 0.64, *SD* = 0.36), Bayes factor = 71 in favor of an effect, as well as the low performance online group (*M* = 0.63, *SD* = 0.33), Bayes factor = 247 in favor of an effect. We found weak evidence of a difference between the high performance online group (*M* = 0.56, *SD* = 0.35) and in-person group, Bayes factor = 2.8 in favor of an effect. Finally, we found only ambiguous evidence that the high performance online group reported less mind-wandering than the other online groups (zero-performance online group, Bayes factor = 1.9 in favor of an effect; low-performance online group, Bayes factor = 2.1 in favor of an effect). These results are illustrated in Figure 7b.

## General Discussion

The goal of the present study was to investigate how multitasking during a complex working memory task affects performance in online and in-person environments. We found that both experimental context and cognitive load had different effects on performance depending on the metric assessed. In Experiment 1, we found that overall, online participants reported mind-wandering much more often than in-person participants, but interestingly and in contrast to expectations, this did not affect their primary working memory performance. Further analyses suggest that while primary working memory performance may be influenced by cognitive load, this may only be evident when comparing single versus dual-task conditions and may not vary with the cognitive load of the secondary task. In Experiment 2, we extended these findings to a different working memory task in a larger online sample, finding again no effect of cognitive load on primary working memory task accuracy, mind-wandering rates, or secondary task performance.

These initial findings provide strong evidence against our prediction that performing the task remotely would lead to impaired working memory performance due to increased mind-wandering that acts as an increased cognitive load. While online participants did report mind-wandering more often than in-person participants, this seemingly did not impact their primary working memory task performance. This may be because cognitive load does not always cause a meaningful change in working memory performance (see also Ricker & Vergauwe, 2020, 2022), and thus, increasing it would not be expected to influence memory accuracy.

We found a large disparity in performance patterns when looking at the secondary task, where online participant performance suffered greatly. However, as depicted in Figures 4 and 6c, this performance impairment was not equal across all participants, with considerable variability in performance on the secondary task in the online groups, suggesting that participants were varying in their engagement with the secondary task. Based on these observations, we pooled the data from the two similar experiments to explore how secondary task engagement affects primary task performance and mind-wandering rates. In the combined analyses, we found that the completely disengaged group, those participants who did not engage with the secondary task at all, performed significantly worse on the working memory task than the in-person and highly engaged online groups. Those participants who were partially engaged with the task (i.e., those who performed poorly on the secondary task but did better than 0%) also performed worse than the in-person and highly engaged online groups and similarly to the disengaged group. Notably, we found that the highly engaged online group performed at least as well, if not better, than the in-person group. All three online groups reported mind-wandering more often than the in-person group. This analysis shows quite clearly that most, but not all, individuals completing the working memory task remotely did show decreased task performance.

The pattern of findings is surprising, in that disengagement with the secondary processing task is typically assumed to result in more resources being made available for primary task completion. This means that lower secondary task engagement should lead to higher primary task performance; however, we observed the opposite. This indicates that the disengaged individuals were not conserving resources for the primary task, but instead failing to engage with the complex span task at the same level they would if completing the task in a laboratory setting. This finding contrasts with a recent study by Uittenhove et al. (2023) comparing working memory performance in a similar complex span task across several different online and in-person sample populations. They found that while in-person student participants had the highest performance overall, students completing the task online performed similarly.

Uittenhove et al. (2023) focused on understanding performance in the primary task, not the secondary task, and enforced high levels of secondary task performance preventing individuals from disengaging with the task without failing to complete the experiment. Although this shows that individuals can perform complex cognitive tasks as well in a remote setting as they do in the lab, our present data show that most participants do not do so when given the autonomy to complete the task as they see fit. Individuals likely vary considerably in how they approach the task when they can choose their level of task engagement with complex cognitive tasks when not in a formal setting. While our present task is a research task, it is likely that the same principle applies to other real-world cognitive tasks such as work and education. Further research is needed that considers how individuals complete complex cognitive tasks when allowed to determine their own levels of engagement if we are to fully understand how individuals typically leverage their cognitive abilities in a remote environment.

Often the role of the secondary task is assumed to only function to worsen primary task performance and secondary task performance is not analyzed or is only used to filter out *poor* participants. However, the processing that occurs during the secondary task has been implicated in long-term memory. McCabe (2008) first reported on the finding that delayed memory performance is better for information originally learned when interleaved with a secondary task compared to alone, although the opposite is true for immediate memory (see also Loaiza et al., 2021; Loaiza & McCabe, 2012, 2013). The initial explanation for this finding was that secondary task processing leads to repeatedly retrieving displaced memory items back into the focus of attention. While the present study does not extend our findings into long-term memory, a clear implication is that lack of secondary task engagement would lead to poorer long-term retention (Cotton, Sandry, & Ricker, 2023). These findings suggest that secondary task processing has important implications for cognition beyond serving as a distractor for a primary task of interest and that individuals engaging in work and education remotely may see lower improvement in learning over time with some cognitive tasks.

In the present study, we also did not find evidence to support one of the leading explanations for the negative effects of multitasking on working memory, cognitive load. According to cognitive load theories, impaired working memory performance during multitasking is due to a reduction in the time available for working memory maintenance (Barrouillet et al., 2004, 2007). However, recent work has cast doubt on the ubiquity of cognitive load effects on working memory performance and suggests that the effect is seen only under specific task conditions (Ricker & Vergauwe, 2020, 2022). The results of both Experiment 1 and Experiment 2 of the present study suggest that while doing two tasks concurrently does impair working memory performance, this was not due to increasing cognitive load as we saw no change in performance when the secondary task cognitive load was increased. Instead, the onset of the secondary task may interfere with some other working memory process, such as consolidation (Jolicøeur & Dell’Acqua, 1998; Nieuwenstein & Wyble, 2014), leading to performance impairment.

The present study has limitations. One limitation is the lack of random assignment to the online and in-person conditions. It is possible that individuals who chose to complete the experiment in-person would be more likely to comply with instructions and be more motivated to perform well on the task, regardless of context. On the other hand, at-home participants could be more likely to disengage, even if they were completing the tasks in-person. However, given the range of performance in the online group, this is an incomplete explanation for our results, as at least some online participants were fully engaged and motivated. Furthermore, by allowing the participants to choose how they completed the experiment, we may have captured a more naturalistic reflection of their cognitive performance in daily life. Still, future research should attempt to replicate the present findings with random assignment to the online and in-person contexts to mitigate possible self-selection biases. A second limitation concerns the frequency of the mind-wandering probe. Previous research has found that relatively infrequent mind-wandering probes (after every 1%–5% of trials) resulted in reliable measurement of individual differences in mind-wandering (Welhaf et al., 2023). While the goal of probing participants’ mind-wandering on every trial was to gain a more fine-grained understanding of the effects of mind-wandering, this high frequency may have influenced the participants’ performance on the task. While the present results do not suggest that it caused participants to remain more on task, given the high rates of mind-wandering reported, future research should vary the frequency of the probes to confirm this. A third limitation is the sample size of the in-person participants compared to the pooled sample online participants. While in-person participants tended to be more engaged with the task, there were a small number of individuals who fell below the 80% accuracy threshold, as seen in Figure 4. Future studies should collect a larger sample size of in-person participants to investigate if a similar pattern is seen in low-performing in-lab participants.

### Conclusion

While most psychology experiments are conducted in a controlled laboratory environment, most real-world cognition takes place under very different circumstances. Recent shifts to remote settings in education and work necessitate a better understanding of how cognition changes across environments. While our initial analyses found no difference in working memory performance between remote and in-person groups, despite different rates of mind-wandering, further analyses suggested that performance impairments are primarily seen in the secondary processing task and overall task engagement. Additionally, while increasing cognitive load did not impact any measure, low participant task engagement corresponded with worse working memory performance and higher rates of mind-wandering. These results underscore the importance of considering several metrics when assessing performance on complex tasks across environments. Furthermore, the present study illustrates that individuals may approach tasks with different strategies, based on many factors, such as task difficulty, motivation, or distraction, especially in self-monitored online contexts.

## Footnotes

^1^
We do not report the results from the Mindfulness Attention Awareness Scale questionnaire as an error in data collection lead to the loss of these data.

^2^
We do not report the results from the Mindfulness Attention Awareness Scale questionnaire as an error in data collection lead to the loss of these data.

## Notes

**Publication Ethics:** These experiments were approved by the Institutional Review Boards at the University of South Dakota and Montclair State University. Informed consent was obtained from all individual participants included in the study.

**Authorship:** All authors contributed to the study conception and design. Experiment programming and data analysis were performed by Kelly Cotton. The first draft of the manuscript was written by Kelly Cotton, and all authors commented on previous versions of the manuscript. All authors read and approved the final manuscript.

**Open Data:** To the best of my ability and knowledge, I have provided all original materials and clear references to all other materials via a stable online repository. Data and analyses scripts are openly available at the project’s Open Science Framework page (https://osf.io/wnvcj; Cotton, Ricker, & Sandry, 2023).
